# EHMTI-0033. The phosphodiesterase 3 inhibitor cilostazol induces migraine-like attacks via cAMP increase

**DOI:** 10.1186/1129-2377-15-S1-A5

**Published:** 2014-09-18

**Authors:** G Song, J Olesen, M Ashina

**Affiliations:** 1 Department of Neurology, Danish Headache Center, Glostrup, Denmark

## Introduction

The initiating mechanisms of migraine attacks are very complex but may involve the cyclic adenosine 3',5'-monophosphate (cAMP) signaling pathway. It is unknown whether intracellular cAMP accumulation induces migraine attacks.

## Aim

To investigate whether administration of cilostazol, which causes cAMP accumulation, may induce migraine attacks.

## Methods

We included 14 migraine patients without aura in a double-blinded, placebo-controlled crossover study. All participants received oral cilostazol or placebo on two separate days. We recorded migraine headache characteristics and associated symptoms using a questionnaire.

## Results

Cilostazol induced delayed migraine-like attacks in 12 patients (out of 14) compared to 2 (out of 14) patients after placebo (P=0.002). The median time to onset for migraine-like attacks was 6 h (range 3-11 h). Patients reported that the attacks mimicked their usual migraine attacks and that cilostazol induced attacks responded to their usual migraine treatment. The median time of medication intake was 6 h (range 4-11).

## Conclusions

The present study suggests that intracellular cAMP accumulation plays a crucial role in migraine induction. This knowledge is a further step in our understanding of the intracellular pathway of migraine initiation.

No conflict of interest.

